# MvAl-MFP: A Multi-Label Classification Method on the Functions of Peptides with Multi-View Active Learning

**DOI:** 10.3390/cimb47080628

**Published:** 2025-08-06

**Authors:** Yuxuan Peng, Jicong Duan, Yuanyuan Dan, Hualong Yu

**Affiliations:** 1 School of Computer, Jiangsu University of Science and Technology, Zhenjiang 212100, China; pengyuxuan@stu.just.edu.cn (Y.P.); jicong_duan@stu.just.edu.cn (J.D.); 2 School of Environmental and Chemical Engineering, Jiangsu University of Science and Technology, Zhenjiang 212100, China

**Keywords:** multifunctional peptide, peptide function prediction, active learning, multi-label learning, query by committee active learning, disagreement metric

## Abstract

The rapid expansion of peptide libraries and the increasing functional diversity of peptides have highlighted the significance of predicting the multifunctional properties of peptides in bioinformatics research. Although supervised learning methods have made advancements, they typically necessitate substantial amounts of labeled data for yielding accurate prediction. This study presents MvAl-MFP, a multi-label active learning approach that incorporates multiple feature views of peptides. This method takes advantage of the natural properties of multi-view representation for amino acid sequences, meets the requirement of the query-by-committee (QBC) active learning paradigm, and further significantly diminishes the requirement for labeled samples while training high-performing models. First, MvAl-MFP generates nine distinct feature views for a few labeled peptide amino acid sequences by considering various peptide characteristics, including amino acid composition, physicochemical properties, evolutionary information, etc. Then, on each independent view, a multi-label classifier is trained based on the labeled samples. Next, a QBC strategy based on the average entropy of predictions across all trained classifiers is adopted to select a specific number of most valuable unlabeled samples to submit them to human experts for labeling by wet-lab experiments. Finally, the aforementioned procedure is iteratively conducted with a constantly expanding labeled set and updating classifiers until it meets the default stopping criterion. The experiments are conducted on a dataset of multifunctional therapeutic peptides annotated with eight functional labels, including anti-bacterial properties, anti-inflammatory properties, anti-cancer properties, etc. The results clearly demonstrate the superiority of the proposed MvAl-MFP method, as it can rapidly improve prediction performance while only labeling a small number of samples. It provides an effective tool for more precise multifunctional peptide prediction while lowering the cost of wet-lab experiments.

## 1. Introduction

Bioactive peptides are short-chain molecules that exert many crucial biological functions. The utilization of these bioactive peptides in medicine has been on the rise due to the rapid expansion of peptide libraries. For instance, antidiabetic peptides help regulate blood sugar levels [1], antimicrobial peptides (AMPs) exhibit anti-bacterial properties by disrupting pathogen cell membranes [2], and neuropeptides (e.g., enkephalins) are pivotal in processes like pain modulation and mood regulation [3]. These diverse functions highlight the potential of bioactive peptides in disease management [4], leading to the continuous growth of the global peptide drug market [5].

Within the extensive peptide family, therapeutic peptides represent a distinct category of drug formulations with multifaceted therapeutic capabilities. In comparison to conventional drugs, therapeutic peptides offer advantages such as cost-effectiveness, low toxicity, and stability when stored at room temperature. Therapeutic peptides have demonstrated significant promise in drug development across various domains including anti-angiogenesis [6], anti-inflammation [7], anti-cancer [8], and other therapeutic areas [9].

Therapeutic peptides encompass a diverse range of bioactive molecules, among which eight categories are of particular interest. Anti-angiogenic peptides (AAPs) suppress pathological neovascularization, especially in tumors and ocular diseases. Anti-bacterial peptides (ABPs) are key innate immune effectors that disrupt bacterial membranes or interfere with intracellular targets. Anti-cancer peptides (ACPs) selectively induce cancer cell death through membrane disruption or apoptosis. Anti-inflammatory peptides (AIPs) downregulate cytokine release and inhibit pathways such as NF-κB and MAPK. Anti-viral peptides (AVPs) block viral entry, fusion, or replication, offering broad-spectrum anti-viral potential. Cell-penetrating peptides (CPPs) cross cell membranes and enable intracellular delivery of various therapeutics. Quorum sensing peptides (QSPs), mainly found in Gram-positive bacteria, mediate intercellular communication via two-component systems and regulate processes like virulence and biofilm formation. Polystyrene-binding peptides (PBPs) show affinity for polystyrene surfaces, aiding ELISA development but also contributing to nonspecific binding in phage display experiments.

The identification and screening of therapeutic peptides pose significant challenges due to the time and resource-intensive nature of traditional experimental methods, such as peptide synthesis and bioactivity testing [10]. Consequently, there is a pressing demand for more efficient and expedited approaches to anticipate and assess the therapeutic attributes of peptides. Recently, machine learning and deep learning technologies have served as powerful tools to tackle this challenge by leveraging peptide sequence data exclusively to forecast their functions [11,12].

Specifically, peptide function prediction typically comprises two main stages: encoding peptides into tensors through suitable feature extraction techniques and subsequently training machine learning or deep learning models to transform peptide function prediction into a multi-label classification task. Efficient feature extraction methods play a crucial role in distinguishing peptides belonging to various functional categories, thereby establishing the groundwork for developing prediction models with high performance. Commonly utilized feature extraction methods include amino acid composition (AAC), dipeptide composition (DPC) [13], pseudo-amino acid composition (PseAAC) [14], composition–transition–distribution (CTD) [15], and position-specific scoring matrix (PSSM) [16], among others.

Various supervised learning techniques have demonstrated significant success in predicting peptide functions, particularly multi-view learning approaches, known for their effectiveness in addressing intricate problems. For instance, PreTP-EL [17] utilizes nine feature views and integrates support vector machines (SVMs) and random forests (RFs) as base classifiers. It enhances classification accuracy by employing genetic algorithms to assign weights to each predictor. TPred-ATMV [18] introduces a method that combines multi-view learning and tensor learning. This approach leverages two discriminative feature representation strategies, namely category information and probability information, to construct a latent subspace that facilitates the seamless integration of information from diverse feature views. Furthermore, Zhao et al. introduced the SCN-MLTPP model [19], which extracts seven feature views and combines them using a stacked capsule network (SCN). SCN-MLTPP effectively mitigates the feature loss issues associated with pooling layers in conventional convolutional neural networks (CNNs).

While multi-view supervised learning techniques demonstrate strong performance in predicting peptide function, they typically depend on substantial quantities of well-annotated data. The limited availability of labeled data in extensive peptide databases poses a notable obstacle. Consequently, there is an urgent need in current research to diminish the requirement for labeling and enhance prediction efficacy [20]. To address this issue, active learning has emerged as an effective and efficient solution. Active learning identifies samples with substantial information content for annotation, thereby reducing the labeling burden and enhancing model accuracy [21].

The essence of active learning is centered on formulating effective query strategies to minimize the number of samples requiring labeling while ensuring efficient model training [22]. In the field of multi-label learning, uncertainty metrics serve as the cornerstone for query strategies, with the most prevalent ones being Maximum Margin Uncertainty (MMU) [23,24], Label Cardinality Inconsistency (LCI) [25], and Example-based Active Learning (EMAL) [26]. MMU primarily estimates sample uncertainty based on global separation boundaries but is sensitive to label noise. LCI evaluates sample uncertainty considering label cardinality, offering simplicity and intuitiveness but potentially lacking in capturing intricate uncertainty. EMAL transforms multi-label issues into multiple single-label sub-issues, integrating uncertainty, which neglects sample diversity, resulting in superfluous sampling and bias. Apart from these conventional uncertainty-driven approaches, some other active learning strategies integrate additional criteria, such as the Integrated Active Learning Strategy [27], Category Vector Inconsistency-based Query Algorithm (CVIRS) [28], and Robust Multi-Label Active Learning (RMLAL) [29]. Nevertheless, these methods often concentrate on optimizing a single dimension, potentially falling short in addressing the intricacies of multi-label scenarios.

In this study, we introduce a query strategy utilizing multi-view disagreement to enhance the performance of models in predicting multifunctional peptides. Barrett et al. [30] previously explored active learning through query by committee (QBC) [31] in the context of single-functional peptide design. They employed One-hot encoding and amino acid numeric coding for inputting data to establish a convolutional neural network query committee with varied hyperparameters. Their experimental results suggested that QBC provided only limited improvements over random sampling. We hypothesize that one possible reason for this is the adoption of insufficient diversity among committee members, as the models were built upon largely similar feature representations of the peptide sequences. In response, we leverage multiple biologically meaningful feature extraction methods to construct heterogeneous feature views, aiming to enhance the diversity among committee members from multiple representation perspectives.

Specifically, the proposed multi-view active learning method for classifying multifunctional therapeutic peptides (MvAl-MFP) first generates nine distinct feature views for a few labeled peptide amino acid sequences by considering various peptide characteristics, including amino acid composition, physicochemical properties, evolutionary information, etc. Then, on each independent view, a multi-label classifier (binary relevance based on logistics regression (LR) [32]) is trained based on the labeled samples. Next, we adopt the QBC strategy based on the average entropy of predictions across all trained classifiers to select a specific number of most valuable unlabeled samples to be submitted to human experts for labeling by wet-lab experiments. Finally, the aforementioned procedure is iteratively conducted with a constantly expanding labeled set and updating classifiers until it meets the default stopping criterion. The experiments are conducted on a subset of a PPTPP dataset [33], which contains all positive samples related to various functional categories. The results in terms of five representative multi-label performance evaluation metrics clearly indicate that adopting a QBC strategy in MvAl-MFP can yield a more superior performance than using a totally random sampling strategy. Also, we observe that aggregating more diverse views helps with promoting the quality of both active learning and ensemble decisions. Additionally, the results have also shown that utilizing some high-quality views can yield an approximate performance to using all views. Combining these observations, we can say that MvAl-MFP provides an effective and efficient tool for multifunctional peptide prediction.

The rest of this paper is organized as follows. Section 2 describes the data used, data processing strategies, feature views, the proposed MvAl-MFP method, and the performance evaluation metrics in detail. In Section 3, extensive experimental results are given and comprehensively analyzed. Finally, Section 4 concludes the findings and contributions of this study.

## 2. Materials and Methods

### 2.1. Dataset Preparation

To assess the efficacy of MvAl-MFP in identifying peptides of high value, experiments were carried out using a dataset of multifunctional therapeutic peptides, PPTPP [33]. This dataset encompasses eight prevalent functional labels for therapeutic peptides in supervised learning, namely AAP, ABP, ACP, AIP, AVP, CPP, QSP, and PBP. All peptides collected underwent the following preprocessing steps: (i) removal of peptides with over 200 amino acids or less than 6 amino acids; (ii) exclusion of peptides containing non-standard residues like “B”, “J”, “O”, “U”, “X”, and “Z”. This preprocessing strategy was adapted from iAMPCN [34], aiming to reduce feature space sparsity and eliminate noise introduced by ambiguous or rare amino acid codes. Following this filtering process, the number of peptides in each functional category was determined, as illustrated in Table 1. As for the distribution of label combinations, it is presented in Table 2.

Given the multifunctional nature of therapeutic peptides in practical applications, only positive examples were retained by excluding negative examples without any one functional category. This approach resulted in a multi-label dataset where each function was considered an independent label. Formally, the label set **Y** can be represented as a two-dimensional matrix with elements being either 1 or 0:

(1) $$Y=\left[\begin{array}{ccc} y_{11} & \ldots & y_{1L}\\ \vdots & \ddots & \vdots \\ y_{N1} & \ldots & y_{NL} \end{array}\right]$$

where *N* stands for the number of peptides in the dataset, and *L* represents the number of functions. Each element in the matrix is defined as follows:

(2) $$y_{i,j}=\left\{\begin{array}{l} 1,\;\mathrm{if}\;\mathrm{the}\;i\mathrm{th}\;\mathrm{peptide}\;\mathrm{holds}\;\mathrm{the}\;j\mathrm{th}\;\mathrm{function}\\ 0,\;\mathrm{if}\;\mathrm{the}\;i\mathrm{th}\;\mathrm{peptide}\;\mathrm{does}\;\mathrm{not}\;\mathrm{hold}\;\mathrm{the}\;j\mathrm{th}\;\mathrm{function} \end{array}\right.$$

We acknowledge that this binary representation is a simplification, as the biological activity of peptides is often concentration dependent. However, since the focus of this study lies in designing an effective active learning framework for multifunctional peptides, this abstraction was adopted to facilitate modeling and evaluation.

To ensure the reliability of the experimental outcomes, a five-fold cross-validation technique was employed on the filtered peptide data. In each iteration of the experiment, 20% of the data was designated as the test set, while the remaining 80% was allocated to the training set. Within the training set, 4% was further randomly segregated as the labeled set, with the remaining 76% forming the unlabeled set. To minimize the impact of random variations, the final results were averaged across five experimental runs.

### 2.2. Construction of Feature Views

Feature representation plays a pivotal role in accurate peptide recognition. Existing research has proposed various methods for representing peptide features, each capturing multidimensional information from distinct perspectives, such as raw sequence data, physicochemical properties, and evolutionary information. Single-feature representation methods often fall short in fully characterizing the properties of peptides. However, by combining multiple feature views, a more comprehensive understanding of a peptide’s multidimensional characteristics can be attained, thereby enhancing peptide recognition performance. Expanding on this concept, this study incorporates it into an active learning framework to improve model performance in predicting multifunctional therapeutic peptides.

To comprehensively capture diverse aspects of peptide characteristics, including sequence composition, physicochemical properties, spatial residue relationships, and evolutionary information, nine following feature views were used in this study: amino acid composition (AAC), dipeptide composition (DPC), composition–transition–distribution (CTD), pseudo-amino acid composition (PseAAC), Distance-Pairs (DP) [35], Distance-based Residue approach (DR) [36], One-hot encoding (Onehot), *k*-Spaced Conjoint Triad (KSCTriad) [37], and PSSM and PSFM cross transformation (PPCT).

AAC, DPC, Onehot, and KSCTriad analyze the peptide’s raw sequence information. Specifically, AAC calculates the frequency of individual amino acids, while DPC computes the occurrence of adjacent amino acid pairs. Onehot represents amino acids as a twenty-dimensional vector in a two-dimensional matrix, with each amino acid encoded at a specific position with a value of 1 and 0 elsewhere. KSCTriad examines longer-range interactions by evaluating amino acid triplets and their spacing, considering amino acids separated by a distance of *k* to provide more comprehensive sequence features.

Both CTD and PseAAC combine raw sequence data with the peptide’s physicochemical properties. Specifically, CTD incorporates factors such as hydrophobicity, normalized van der Waals volume, and polarity by categorizing the 20 amino acids into three groups based on these properties. It then calculates the frequency of occurrence within each group, transition information between groups, and their distribution throughout the sequence. PseAAC enhances AAC by introducing amino acid physicochemical attributes and sequence dependency features. It employs normalization and correlation functions to analyze the relationships among amino acid properties effectively.

DP and DR both analyze the sequential information of amino acid pairs in peptides. DP characterizes the structural aspects of peptide sequences by assessing the relative positions of amino acid pairs, in conjunction with secondary structure and physicochemical properties. On the other hand, DR quantifies the occurrence frequency of amino acid pairs within a defined distance range, thus capturing sequence order and associated characteristics.

As for PPCT, it merges a position-specific scoring matrix (PSSM) and position-specific frequency matrix (PSFM) to depict peptide sequences and encompass evolutionary insights. Both PSSM and PSFM are derived from multiple sequence alignments conducted using PSI-BLAST [38]. PSSM reflects the occurrence frequency of amino acids at specific positions and their mutational tendencies, while PSFM considers the distribution of amino acids at diverse positions in protein sequences. By integrating data from both matrices and addressing their dimensional disparities, PPCT generates a standardized 2000-dimensional vector.

Specifically, the dimension information of the nine feature views used are listed in Table 3.

### 2.3. MvAl-MFP Algorithm

**Figure 1:**
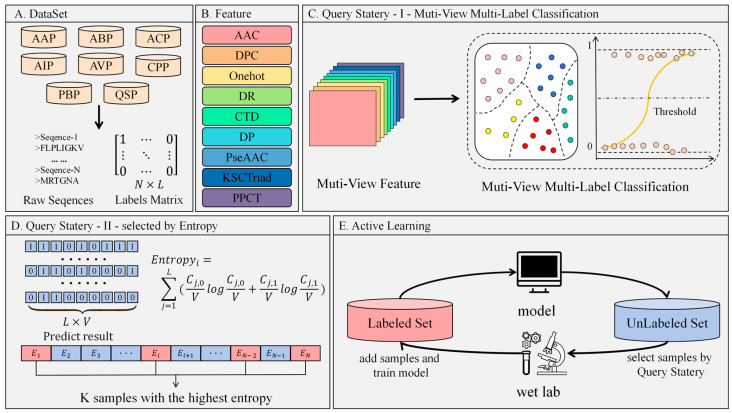
The procedure of the MvAl-MFP algorithm: (**A**) the construction of the multifunctional peptides dataset; (**B**) multi-views representation; (**C**) multi-view multi-label classification procedure; (**D**) entropy-based QBC query strategy; (**E**) active learning iteration procedure.

In the proposed MvAl-MFP algorithm, the active learning process comprises four key components: the labeled set ($S_{L}$), the unlabeled set ($S_{U}$), the human expert (H), and the query model (C), as illustrated in Figure 1. Initially, a model M is trained using the labeled set, and its performance is assessed on the unlabeled set. Subsequently, the most informative samples are chosen for annotation by human experts. In the context of peptide-related studies, experts perform wet-lab experiments to assess the functions of the selected peptides, assign labels indicating their true functions, and incorporate them into the labeled set, facilitating iterative cycles.

To evaluate the efficacy of active learning, we incorporate an independent test set. During each iteration, the model *M* undergoes training on the labeled set and subsequent evaluation on the independent test set. Let D denote a performance evaluation metric, where a higher D indicates superior model performance. Specifically, $D_{i}$ denotes the performance of the model *M* in the *i*th active learning iteration, while $F({i,D}_{i})$ characterizes the relationship between model performance and each iteration. The primary objective of active learning is to devise an optimal query strategy that enhances the model’s performance, thereby facilitating the rapid growth of $F({i,D}_{i})$ with each successive iteration.

Specifically, $S_{L}=\left\{{(O}_{1},Y_{1}\right),\left(O_{2},Y_{2}\right)\ldots (O_{N_{L}},Y_{N_{L}})\}$ and $S_{U}=\{O_{1},O_{2}\ldots O_{N_{u}}\}$ are defined as the labeled and unlabeled set of peptides, respectively, where $O_{i}$ denotes the *i*th peptide sequence, and $N_{L}$ and $N_{U}$ represent the number of peptides in the labeled and unlabeled sets, respectively. The vector $Y_{i}$ denotes the function labels, which are defined according to Equations (1) and (2).

In each iteration, the query model is trained using the current labeled set $S_{L}$. The initial step of the query model involves the feature representation from original peptide sequences through various feature representation methods (feature views), yielding $\left\{B_{1},B_{2}\ldots B_{V}\right\}$, where *V* denotes the number of feature views. Each feature view is depicted as a two-dimensional matrix, with each line indicating a sample (i.e., a peptide) and each column representing a feature. Specifically, the lengths of the columns for the nine feature views employed in this research are detailed in Table 2. Subsequently, a multi-label classification model is constructed on each feature view, resulting in $\left\{M_{1},M_{2}\ldots M_{V}\right\}$. Each model is then utilized to predict the labels of each sample in the unlabeled set $S_{U}$, generating a prediction matrix $Y^{\prime }$ as defined in Equation (3):

(3) $$Y^{\prime }=\left[\begin{array}{ccc} y_{11}^{\prime } & \ldots & y_{1L}^{\prime }\\ \vdots & \ddots & \vdots \\ y_{V1}^{\prime } & \ldots & y_{VL}^{\prime } \end{array}\right]$$

where $y_{ij}^{\prime }$ denotes that for a specific sample, its *j*th label is predicted by the *i*th model $M_{i}$, which is either 0 or 1.

As we know, the QBC active learning strategy always calculates the disagreement level among multiple models and then selects several samples with the highest disagreement levels to query labels from human experts. In this study, we use total voting entropy across all labels to calculate the disagreement level of an unlabeled sample:

(4) $${Entropy}_{i}=\sum_{j=1}^{L}(\frac{C_{j,0}}{V}\log\frac{C_{j,0}}{V}+\frac{C_{j,\;1}}{V}\log\frac{C_{j,\;1}}{V})$$

where $C_{j,0}$ and $C_{j,\;1}$ denote the voting number for 0 and 1 on the *j*th label of the *i*th unlabeled sample, respectively. Then, all unlabeled samples are ranked by voting entropy in descending order, and some high-order samples are selected to be submitted to human experts for labeling by wet-lab experiments. The selected samples are as follows:

(5) $$S_{U}^{*}=a\mathrm{rg}\max\left(Entropy\left(S_{U}\right)\right)$$

where $S_{U}^{*}=\left\{s_{1},s_{2},\ldots ,s_{\lambda }\right\}$ and λ denotes the number of samples selected in each iteration. Next, after these λ samples are labeled by human experts, they are used to extend $S_{U}$ and further update $\left\{M_{1},M_{2}\ldots M_{V}\right\}$ to prepare the next iteration.

Finally, after active learning satisfies the pre-setting stopping criterion, V final models are used to make a decision for future samples as follows:

(6) $$h^{\prime }=sign\left(\sum_{i=1}^{V}{\;\;\;\;P}_{i}\right)$$

where $P_{i}$ denotes the likelihood of the predicted sample by the *i*th model, $sign\left(\cdot \right)$ is the sigmoid function, and $h^{\prime }$ represents the corresponding predicted label.

The pseudo-code of the proposed MvAl-MFP algorithm (Algorithm 1) is described as follows:

**Algorithm 1**: MvAl-MFP**Input**: The initial labeled set $S_{L}$, the initial unlabeled set $S_{U}$, the number of labels *L*, the human expert H, the multi-label learning model M, the number of feature representation methods V, feature representation methods $\left\{B_{1},B_{2}\ldots B_{V}\right\}$, the number of selected unlabeled samples in each iteration λ. **Output**: Ensemble multi-label classifiers {$M_{1},M_{2}\ldots M_{V}$} **Procedure**: **For** *i* = 1:*V*  Transform $S_{L}$ and $S_{U}$ by $B_{i}$;  Train the initial classifier $M_{i}$ on the transformed $S_{L}$; **End for** **Repeat** until satisfying the presetting stopping criterion    **For** *i* = 1:|$S_{U}$|     Generate $Y_{i}^{\prime }$ by adopting {$M_{1},M_{2}\ldots M_{V}$} to predict its *L* labels (see Equation (3));     Calculate its voting entropy ${Entropy}_{i}$ by Equation (4);  **End for**  Rank voting entropies of all unlabeled samples in descending order, and select Top-*λ* ones into $S_{U}^{*}=\left\{s_{1},s_{2},\ldots ,s_{\lambda }\right\}$ by Equation (5);  Submit $S_{U}^{*}$ to *H* for acquiring real labels by wet-lab experiments;  Add $S_{U}^{*}=\left\{s_{1},s_{2},\ldots ,s_{\lambda }\right\}$ with real labels into $S_{L}$;  Remove $S_{U}^{*}=\left\{s_{1},s_{2},\ldots ,s_{\lambda }\right\}$ from $S_{U}$;  Update {$M_{1},M_{2}\ldots M_{V}$} by using the extended $S_{L}$;Output the final {$M_{1},M_{2}\ldots M_{V}$} and make decision for future unseen samples by them (see Equation (6)).

The main computational cost of MvAl-MFP arises from repeatedly training multi-label classifiers on the labeled set and predicting on the unlabeled set. In practice, the classification module can be replaced with any suitable algorithm. In our experiments, we use the binary relevance strategy with logistic regression as the base learner. Each iteration involves training V×L classifiers, yielding a training complexity of $O\left(\sum_{i=1}^{V}\sum_{j=1}^{L}t_{i,j}\cdot n_{j}\cdot d_{i}\right)$, where $d_{i}$ is the feature dimension of view i, $n_{j}$ is the number of labeled samples with label j, and $t_{i,j}$ is the number of training iterations.

To compute disagreement scores, each classifier makes predictions on the unlabeled set, incurring $O\left(\sum_{i=1}^{V}\sum_{j=1}^{L}m_{j}\cdot d_{i}\right)$ where $m_{j}$ is the number of unlabeled samples relevant to label j. Given τ total iterations, the overall time complexity is $O\left(\tau \cdot \sum_{i=1}^{V}\sum_{j=1}^{L}\left(t_{i,j}\cdot n_{j}\cdot d_{i}+m_{j}\cdot d_{i}\right)\right)$.

### 2.4. Performance Evaluation

To thoroughly assess the performance of MvAl-MFP, we employed five popular metrics in multi-label classification: Macro-AUC (Macro Area Under the ROC Curve), Micro-AUC (Micro Area Under the ROC Curve), APS (average precision score), One Error, and Ranking Loss [39,40]. We know that AUC is widely used to evaluate the model’s predictive accuracy across various classification thresholds. Specifically, in multi-label learning, Macro-AUC calculates the average AUC for each label to provide an overall performance assessment, while Micro-AUC is determined by flattening all true labels and predicted probabilities for all samples and then calculating the AUC value. APS measures the model’s precision for individual labels by calculating the average precision (AP) for each label and subsequently averaging these values across all labels. AP is obtained from the precision–recall curve of each label, considering the precision variances across different thresholds. One Error determines if the top-ranked predicted label matches any of the true labels. Failure to match any true label is considered an error. Ranking Loss assesses the ratio of inversely ranked label pairs. In a label pair scenario involving labels *j* and *k*, if label *j* is predicted to have a higher rank than the label *k*, but in reality, label *k* holds a higher true rank than label *j*, this results in a ranking loss.

(7) $$Macro\;AUC=\frac{1}{L}\sum_{j=1}^{L}\frac{\left|\left\{\left(x_{i},x_{k}\right)|f\left(x_{i},y_{j}\right)\geq f\left(x_{k},y_{j}\right),\left(x_{i},x_{k}\right)\in Z_{j}^{+}\times Z_{j}^{-}\right\}\right|}{\left|Z_{j}^{+}\right|\left|Z_{j}^{-}\right|}\;$$

(8) $$Micro\;AUC=\frac{\left|\left\{\left(x_{i},x_{k},y_{i},y_{k}\right)|f\left(x_{i},y_{i}\right)\geq f\left(x_{k},y_{k}\right),\;\;\left(x_{i},y_{i}\right)\in S^{+},\;\;\left(x_{k},y_{k}\right)\in S^{-}\right\}\right|}{\left|S^{+}\right|\left|S^{-}\right|}$$

(9) $$APS=\frac{1}{N}\sum_{i=1}^{N}\frac{1}{\left|Y_{i}\right|}\sum_{y\in Y_{i}}\frac{\left|\left\{y^{\prime }|rank_{f}\left(x_{i},y^{\prime }\right)\leq rank_{f}\left(x_{i},y\right),\;\;y^{\prime }\in Y_{i}\right\}\right|}{rank_{f}\left(x_{i},\;y\right)}\;$$

(10) $$One\;Error=\frac{1}{N}\sum_{i=1}^{N}\left[\;\left[\arg\max f\left(x_{i},y\right)\right]\notin Y_{i}\right]$$

(11) $$Ranking\;Loss=\frac{1}{N}\sum_{i=1}^{N}\frac{1}{\left|Y_{i}\right||\bar{Y_{i}}|}\left|\left\{\left(y,y^{\prime }\right)|f\left(x_{i},y\right)\leq f\left(x_{i},y^{\prime }\right)\in Y_{i}\times \bar{Y_{i}}\right\}\right|$$

Here, *N* represents the total number of samples, and *L* denotes the number of labels. $f\left(x,y_{j}\right)$ is the predicted probability for the label *j* of the sample *x*. $Z_{j}^{+}=\left\{x_{i}|y_{j}\in Y_{i},\;1\;\leq i\leq \;L\right\}$ and $Z_{j}^{-}=\left\{x_{i}|y_{j}\notin Y_{i},\;1\;\leq i\leq \;L\right\}$ correspond to the set of test instances with (without) label $y_{j}$. $S^{+}=\left\{\left(x_{i},y\right)|y\in Y_{i},\;1\;\leq i\leq \;L\right\}$ and $S^{-}=\left\{\left(x_{i},y\right)|y\notin Y_{i},\;1\;\leq i\leq \;L\right\}$ correspond to the set of relevant (irrelevant) instance–label pairs. Specifically, $rank_{f}\left(x_{i},y\right)$ denotes the model’s predicted score for each label. The ranking of relevant labels $Y_{i}$ is determined by their scores, with labels ranked higher indicating more confident predictions by the model. It is noteworthy that for the first three evaluation metrics, the higher the better, while for *One Error* and *Ranking Loss*, the lower the better.

### 2.5. Experimental Environment

All experiments were run on an Intel (R) Core (TM) i5-14600KF 3.50 GHz 14-core CPU with 32 GB RAM using Python 3.12 running environment. All the algorithm flows in this experiment are mainly implemented based on the numpy and scikit-learning libraries and do not involve GPU operations.

## 3. Results and Discussions

### 3.1. Comparison with Random Query Strategy and Random-k-View Methods

First, to verify the effectiveness of the proposed QBC query strategy, we compared it to the random query strategy. In addition, we were curious about the impact of the number of feature views on the performance of the proposed MvAl-MFP algorithm; thus, we also made a comparison between adopting full views (all nine views) and the following number of random views: three views (DP, KSCTriad, PseAAC), five views (AAC, CTD, DP, DPC, PPCT), and seven views (AAC, CTD, DP, DPC, KSCTriad, Onehot, PseAAC). The number of unlabeled samples selected in each iteration λ was empirically set to 30. A smaller value would significantly prolong the experimental process, while a larger value may introduce too many samples with low disagreement. Specifically, we adopted the binary relevance based on logistics regression (LR) [32] as the default multi-label classifier due to its simplicity and rapid training speed. In practical applications, users are encouraged to replace it using any one multi-label classifier. To reduce experimental time, the process is terminated when the number of labeled samples reaches 65% of the initial unlabeled pool. This threshold is informed by empirical findings in active learning literature, where model performance typically converges to an optimal level once 60% to 70% of the data have been labeled.

**Figure 2:**
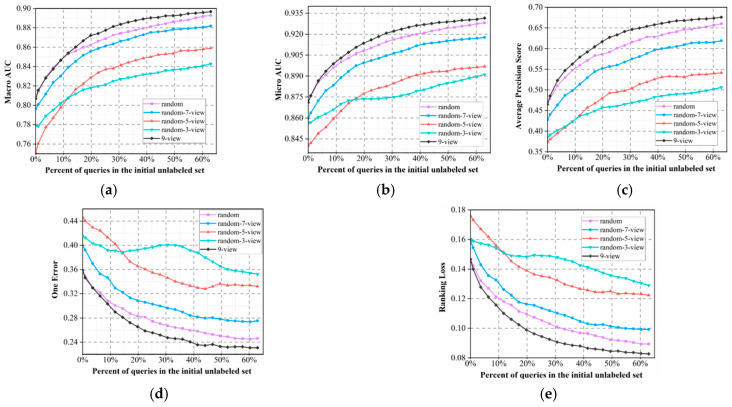
Performance variation curves of the MvAl-MFP algorithm with full views (nine views), three random views, five random views, seven random views, and a random query based on nine views, under the following metrics: (**a**) Macro-AUC, (**b**) Micro-AUC, (**c**) APS, (**d**) One Error, and (**e**) Ranking Loss.

Further, to avoid the impact of random factors, we randomly divided the dataset into labeled, unlabeled, and test sets ten times and used their average as the results, which are presented in Figure 2.

In Figure 2, we observe that although both the nine-view and random methods adopt the same nine views to constitute the QBC model, there is discrepancy in their performances in terms of the five different evaluation metrics. Specifically, the proposed nine-view method utilizes voting entropy to select those most informative samples for labeling, which helps to rapidly enhance the quality of learning models. In contrast, a random query tends to waste lots of queries for labeling potentially nonsignificant samples, further adding to the experimental burden of human experts. Results show that our multi-view model outperforms the QBC approach proposed by Barrett et al. [30], effectively overcoming its limitations. We believe this is due to the use of diverse feature views that capture the different biological properties of peptides, such as sequence composition, evolutionary profiles, and physicochemical characteristics. Unlike their method, which relied on similar input features with varied hyperparameters, our approach builds committee diversity at the data level. This leads to stronger prediction disagreement and improves the effectiveness of active learning in multi-label peptide prediction.

Additionally, the curves in Figure 2 present a clear rule, that is, when we randomly select views to constitute QBC, its performance can be significantly improved with the number of selected views *k*. It can be seen that nine views obviously perform better than seven random views, which significantly outperform two others, and three random views perform the worst. We believe that the reasons are two-fold. First, adopting more views often tends to provide more accurate uncertainty estimation for extracting more valuable unlabeled samples, further accelerating improvement in the quality of learning models. The other reason lies in the fact that, in ensemble learning, when we do not consider the disturbance of other factors, aggregating more base learners always tends to yield better results. This is relevant to the basic theory of ensemble learning [41], i.e., suppose *V* learners in an ensemble share a common error rate ϵ, the expected ensemble error rate can then be represented as follows:

(12) $$P\left(G\left(x\right)\neq f\left(x\right)\right)=\sum_{k=0}^{\left\lfloor \frac{V}{2}\right\rfloor }C_{V}^{k}{\left(1-\epsilon \right)}^{k}\epsilon^{V-k}\leq \exp\left(-\frac{1}{2}V{\left(1-2\epsilon \right)}^{2}\right)$$

It is clear that the second reason is more significant, as the nine-views-based random query performs obviously better than several other random-*k*-view strategies.

Finally, as shown in Figure 2, the performance improves rapidly at the beginning and stabilizes after querying approximately 40% of the unlabeled pool, indicating that most informative samples are selected early. This early convergence demonstrates the effectiveness of our method in achieving high accuracy with reduced labeling effort and experimental cost.

### 3.2. Balance Test About Function Selection

**Figure 3:**
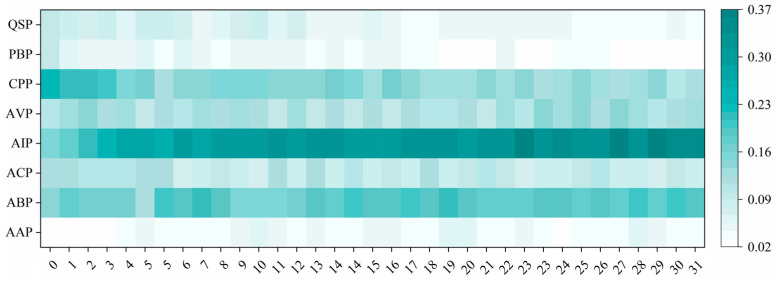
Statistical results on the proportion of selected samples covering each function label in each iteration of active learning, where the horizontal axis denotes the cumulative proportion of labeled and unlabeled samples, the vertical axis indicates the function category, and the color bar shows the proportion value.

Next, to further verify the impartiality of the proposed MvAl-MFP algorithm by equally focusing on every function label, we made a balance test about its function selection during active learning. Specifically, in each iteration, we tracked and conducted statistics about the proportion of selected samples covering each function category. Figure 3 presents the statistical results.

In Figure 3, it can be observed that although the proposed MvAl-MFP algorithm tends to select significantly more samples from those frequent function categories, e.g., AIP and ABP, during the entirety of active learning, it can still provide a relatively balanced selection for samples belonging to each function category in the initial phase of active learning. We believe that this can be attributed to the adoption of average voting entropy across all function labels (see Equation (4)), guaranteeing that the uncertainty of each label on any one specific sample can be treated equivalently. Surely, we have to admit that when active learning is persistently conducted, there exists a potential risk of class imbalance [42,43] for classifiers, which tends to push classifiers to focus more on those frequent function categories but ignore some other scare ones. However, this issue is not easy to solve. In practice, it is often unclear whether the reduced focus on scarce categories is caused by label imbalance or because the model has already achieved good performance on those labels. In future work, we will explore integrating multi-label imbalance handling techniques into the active learning process to better address this challenge.

### 3.3. View Selection and Optimal View Combinations

Further, we are curious about two following questions: (1) On which views do learning models perform better? (2) Can aggregating several high-quality views yield better or comparable performance than/to integrating all views? To answer these two questions, a group of new experiments was designed.

**Figure 4:**
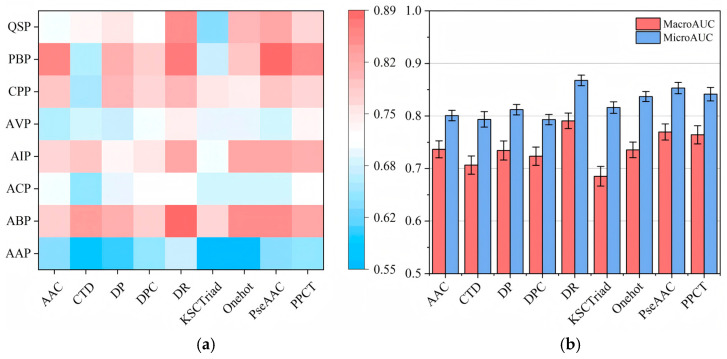
(**a**) The AUC value of each function category on each independent view and (**b**) the Macro-AUC and Micro-AUC values on each view.

First, we tried to answer the first question by measuring the AUC (area under the ROC curve) value of each function category on each independent view, and the Macro-AUC and Micro-AUC values on each view, respectively. In particular, we only used the initial labeled set to train the LR classifier and provided results measured on the test set. The results are presented in Figure 4.

From the results illustrated in Figure 4, it is not difficult to observe that each view can provide different strengths of support for predicting different function categories, and on different views, it can construct prediction models with discrepant qualities. Obviously, the models constructed on DR, PseAAC, and PPCT views perform significantly better than the models trained on several other independent views. In contrast, the models trained on AAC, CTD, and DPC views perform worst. This phenomenon can be understood as the inferior views always very one-sidedly describing the original amino acid sequences, e.g., AAC only counts the frequency of each amino acid but fails to provide more intricate features to describe the structural information and physicochemical properties of peptide sequences. According to these results, we can say that under different views, learning models perform differently.

**Figure 5:**
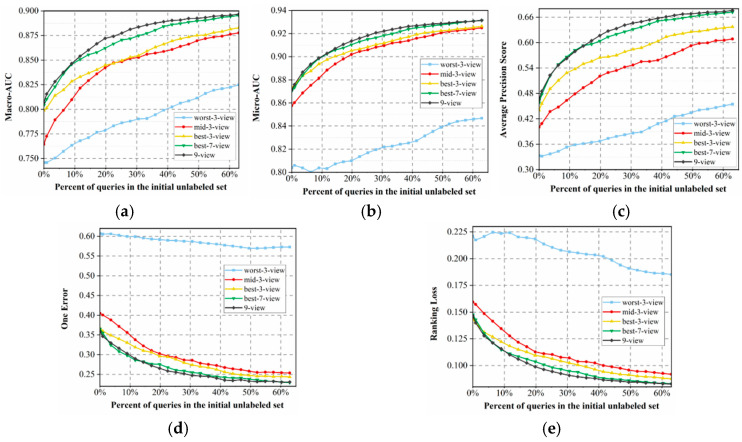
Performance variation curves of various view combinations, including the nine-view approach, best-3-view, mid-3-view, worst-3-view, and best-7-view, under the following metrics: (**a**) Macro-AUC, (**b**) Micro-AUC, (**c**) APS, (**d**) One Error, and (**e**) Ranking Loss.

Next, we wish to answer the second question by integrating views with different qualities into our active learning framework and comparing them with the proposed nine-view approach. Specifically, we divided the view combinations into three basic groups: best-3-view (DR, PseAAC, and PPCT), mid-3-view (DP, KSCTriad, and Onehot), and worst-3-view (AAC, CTD, and DPC) to observe whether integrating high-quality views can yield better performance. In addition, we also used best-7-view to compare with the nine-view approach to observe whether removing poor views helps with enhancing the performance of our proposed method. The performance variation curves of these view combinations are presented in Figure 5.

In Figure 5, the results show that integrating high-quality views can significantly enhance the performance of our proposed active learning framework. Specifically, worst-3-view performs obviously worse than several other view combinations, best-3-view performs a little better than mid-3-view, and best-7-view presents quite an approximated performance compared to the nine-view approach. It illustrates that only employing poor views can tremendously lower the quality of our proposed MvAl-MFP algorithm, while integrating a small number of poor views into high-quality views tends to enhance the quality of MvAl-MFP. We believe that it can be explained by the “bias-variance decomposition” theory [44], which indicates that the generalization error *E* of an ensemble classifier can be mathematically represented as follows:

(13) $$E=\bar{E}-\bar{A}$$

where $\bar{E}$ and $\bar{A}$ represent the average generalization error and average diversity of all models in the ensemble, respectively. This means that a successful ensemble learning model should simultaneously satisfy the following two conditions: each individual learner should be as accurate as possible and, among different learners, they should be as diverse as possible. This explains our experimental findings well.

The comparable performance between the best-7-view combination and the full nine-view setting suggests that removing certain high-dimensional yet low-quality views can retain near-optimal performance while reducing computational cost. This insight provides a practical guideline for selecting efficient view combinations in real-world applications.

Finally, we present the baseline results yielded by various view combinations with training on both labeled and unlabeled datasets in Table 4. The results strengthen the aforementioned conclusions.

### 3.4. Compared with Other Multi-Label Active Learning Methods

To further demonstrate the superiority of the proposed method, we compared MvAl-MFP with two representative multi-label active learning approaches: LCI and MMU. As these methods are not inherently designed for multi-view feature inputs, we concatenated all feature views to construct a unified feature space for them. To ensure fair comparison focused on query strategies, all methods adopted the same binary relevance classifier using logistic regression.

Since the area under the performance curves can intuitively reflect the overall effectiveness of active learning algorithms, we computed the area under the curves of Macro-AUC, Micro-AUC, APS, One Error, and Ranking Loss with respect to the number of labeled samples. The results are summarized in Table 5.

The results show that both MvAl-MFP and MMU outperform the random selection baseline across all five metrics, demonstrating the effectiveness of their active learning strategies. In contrast, LCI performs slightly worse than random sampling, indicating a potential negative effect in this task. This may be due to the fact that LCI identifies outliers based on deviations in the number of predicted positive labels. In our dataset, however, most peptides possess only one or two functional labels, resulting in limited uncertainty variation across samples and thereby reducing the effectiveness of LCI.

MvAl-MFP outperforms MMU, which we attribute to its multi-view joint prediction strategy. MMU relies on constructing a global decision boundary, often using SVMs, which requires high-quality feature spaces. However, bioinformatics-based peptide features vary significantly in dimensionality; for instance, PPCT has 2000 dimensions while PseAAC has only 25. After concatenation, high-dimensional features may dominate predictions, overshadowing lower-dimensional yet informative views. Achieving optimal performance with single-view-based methods like MMU may require more elaborate feature engineering, increasing both development complexity and computational cost.

In summary, MvAl-MFP not only accounts for the label sparsity common in multifunctional peptide data but also integrates diverse bioinformatic feature representations effectively. This contributes to both improved performance and better interpretability, making MvAl-MFP a more efficient and robust choice compared to existing multi-label active learning methods.

## 4. Conclusions

In this study, we take advantage of the natural properties of representation of multiple views of peptide sequences to design a multi-view multi-label active learning algorithm called MvAl-MFP for recognizing multifunctional peptides. Specifically, MvAl-MFP profits from the idea of the QBC active learning paradigm to train an individual classifier on each view and then utilizes voting entropy across all labels to evaluate the uncertainty level of each unlabeled sample. By iteratively learning those significant samples, the learning models can rapidly promote the recognition rate of multifunctional peptides and simultaneously significantly reduce the consumption of wet-lab experiments. We verified its effectiveness and superiority on a cleaned multifunctional therapeutic peptides dataset. In addition, the experimental results indicate that simultaneously selecting high-quality views and increasing diversity among views help enhance the quality of MvAl-MFP.

Our current study still faces several limitations. These include the inability to identify peptides with no known functions, the risk of class imbalance as iterations progress, and the lack of label correlation consideration during sample selection. In future work, we plan to incorporate techniques from class imbalance learning and multi-label classification to address these challenges.

## Figures and Tables

**Table 1:** The number of samples in each function category.

Function	Abbreviation	Number of Samples
Anti-angiogenic peptides	AAP	135
Anti-bacterial peptide	ABP	981
Anti-cancer peptides	ACP	367
Anti-inflammatory peptides	AIP	1678
Anti-viral peptides	AVP	604
Cell-penetrating peptides	CPP	462
Polystyrene surface binding peptides	PBP	104
Quorum sensing peptides	QSP	179

**Table 2:** The distribution of label combinations.

Number of Labels Contained in One Sample	Number of Samples
1	4279
2	90
3	17

**Table 3:** The dimension information of the nine feature views used.

Name of Feature View	Dimension
AAC	20
CTD	273
DP	602
DPC	400
DR	1220
KSCTraid	343
Onehot	4200
PseAAC	25
PPCT	2000

**Table 4:** Baseline results yielded by various view combinations with training on both labeled and unlabeled datasets, where on each metric the best result has been highlighted in bold, and the values presented are calculated according to Formulae (7)–(11).

View Combination	Macro AUC	Micro AUC	APS	ONE Error	Ranking Loss
Nine views	**0.903 ± 0.009**	**0.935 ± 0.006**	**0.680 ± 0.018**	**0.232 ± 0.013**	**0.082 ± 0.006**
Random-3-view	0.869 ± 0.010	0.910 ± 0.006	0.560 ± 0.015	0.325 ± 0.015	0.108 ± 0.007
Random-5-view	0.874 ± 0.009	0.908 ± 0.007	0.580 ± 0.018	0.323 ± 0.015	0.111 ± 0.007
Random-7-view	0.891 ± 0.009	0.922 ± 0.006	0.631 ± 0.016	0.280 ± 0.014	0.095 ± 0.006
Worst-3-view	0.841 ± 0.010	0.875 ± 0.006	0.493 ± 0.020	0.425 ± 0.016	0.143 ± 0.006
Mid-3-view	0.888 ± 0.010	0.932 ± 0.006	0.632 ± 0.015	0.245 ± 0.015	0.086 ± 0.006
Best-3-view	0.892 ± 0.008	0.932 ± 0.005	0.652 ± 0.018	0.237 ± 0.013	**0.082 ± 0.006**
Best-7-view	0.902 ± 0.010	**0.935 ± 0.006**	0.677 ± 0.017	**0.232 ± 0.014**	**0.082 ± 0.007**

**Table 5:** Area under performance variation curves of various multi-label active learning methods, including MvAl-MFP with nine views, LCI, MMU, and random, where under each metric the best result has been highlighted in bold.

Method Name	Macro-AUC	Micro-AUC	APS	ONE Error	Ranking Loss
Random	0.5472 ± 0.0055	0.5749 ± 0.0034	0.3845 ± 0.0081	0.1658 ± 0.0100	0.0644 ± 0.0037
LCI	0.5463 ± 0.0062	0.5735 ± 0.0040	0.3757 ± 0.0089	0.1714 ± 0.0100	0.0662 ± 0.0047
MMU	0.5491 ± 0.0057	0.5756 ± 0.0036	0.3820 ± 0.0094	0.1711 ± 0.0090	0.0651 ± 0.0040
MvAl-MFP	**0.5507 ± 0.0058**	**0.5772 ± 0.0034**	**0.3923 ± 0.0091**	**0.1633 ± 0.0078**	**0.0612 ± 0.0037**

## Data Availability

The cleaned multifunctional peptides dataset and the source code of the proposed MvAl-MFP algorithm can be downloaded at: https://github.com/PengYX-7223/MvAl-MFP (accessed on 30 July 2025).

## Abbreviations

The following abbreviations are used in this manuscript:

AAC	Amino acid composition
AAP	Anti-angiogenic peptides
ABP	Anti-bacterial peptide
ACP	Anti-cancer peptides
AIP	Anti-inflammatory peptides
APS	Average precision score
AUC	Area Under the ROC Curve
AVP	Anti-viral peptides
CPP	Cell-penetrating peptides
CTD	Composition–transition–distribution
CVIRS	Category Vector Inconsistency-based Query Algorithm
DP	Distance-Pairs
DPC	Dipeptide composition
DR	Distance-based Residue approach
EMAL	Example-based Active Learning
KSCTriad	*k*-Spaced Conjoint Triad
LCI	Label Cardinality Inconsistency
LR	Logistics regression
Macro AUC	Macro Area Under the ROC Curve
Micro AUC	Micro Area Under the ROC Curve
MMU	Maximum Margin Uncertainty
MTP	Multifunctional peptides
Onehot	One-hot encoding
PBP	Polystyrene surface binding peptides
PPCT	PSSM and PSFM cross transformation
PseAAC	Pseudo-Amino Acid Composition
PSFM	Position-Specific Frequency Matrix
PSSM	Position-Specific Scoring Matrix
QBC	Query by committee
QSP	Quorum sensing peptides
RF	Random forests
RMLAL	Robust Multi-Label Active Learning
SVM	Support vector machine
